# Shining a light on species coexistence: visual traits drive bumblebee communities

**DOI:** 10.1098/rspb.2022.2548

**Published:** 2023-04-12

**Authors:** Océane Bartholomée, Ciara Dwyer, Pierre Tichit, Paul Caplat, Emily Baird, Henrik G. Smith

**Affiliations:** ^1^ Centre for Environmental and Climate Science, Lund University, Lund 22362, Sweden; ^2^ Department of Biology, Lund University, Lund 22362, Sweden; ^3^ Department of Zoology, Stockholm University, Stockholm 10691, Sweden; ^4^ Institute for Global Food Security, School of Biological Sciences, Queen's University Belfast, BT9 5DL UK

**Keywords:** bilberry, bumblebees, microhabitat niche partitioning, sensory traits, vision

## Abstract

Local coexistence of bees has been explained by flower resource partitioning, but coexisting bumblebee species often have strongly overlapping diets. We investigated if light microhabitat niche separation, underpinned by visual traits, could serve as an alternative mechanism underlying local coexistence of bumblebee species. To this end, we focused on a homogeneous flower resource—bilberry—in a heterogeneous light environment—hemi-boreal forests. We found that bumblebee communities segregated along a gradient of light intensity. The community-weighted mean of the eye parameter—a metric measuring the compromise between light sensitivity and visual resolution—decreased with light intensity, showing a higher investment in light sensitivity of communities observed in darker conditions. This pattern was consistent at the species level. In general, species with higher eye parameter (larger investment in light sensitivity) foraged in dimmer light than those with a lower eye parameter (higher investment in visual resolution). Moreover, species realized niche optimum was linearly related to their eye parameter. These results suggest microhabitat niche partitioning to be a potential mechanism underpinning bumblebee species coexistence. This study highlights the importance of considering sensory traits when studying pollinator habitat use and their ability to cope with changing environments.

## 1. Introduction

Understanding mechanisms that underpin species coexistence remains a central theme of ecology. Coexistence has traditionally been explained by niche partitioning, i.e. that species partition resources needed for survival and reproduction in space or time [1,2]. Local coexistence of bees is an iconic example of niche partitioning that has been explained by foraging specialization on flowers [3,4] based on trait matching [5,6]. However, coexisting bee species often demonstrate considerable overlap in their choice of flowers [7], suggesting that we still lack knowledge of what mechanisms underpins local coexistence. This may constrain our ability to understand what drives current losses of bee diversity.

Spatial [8,9] and temporal [10,11] microhabitat selection based on light, temperature and wind conditions have been suggested as an alternative or complementary mechanism to explain niche partitioning in bees. According to this microhabitat hypothesis, bee species with differences in traits associated with foraging could avoid competition by exploiting flower resources in local microhabitats or at times of day to which they are best adapted [11–13]. An extreme example of light-based niche separation is the evolution of nocturnal activity in some bees, a separation reflected in the visual specializations of nocturnal versus diurnal species [13,14]. Niche partitioning associated with visual trait and visual niche variations has also been demonstrated in several diurnal insect groups, including damselflies [15], *Drosophila* [16] and tropical butterflies [17]. As diurnal bees rely primarily on vision to control flight [18,19] and discriminate flowers [20], it is possible that similar partitioning across visual niches could also explain bumblebee species coexistence. While it is known that bumblebees (all of which are considered diurnal) can be active during relatively dark conditions [21,22], light-related niche partitioning remains unexplored as a potential explanation for bumblebee species coexistence, likely due to the fact that surveys of pollinator abundance are typically restricted to the brightest of daylight conditions (e.g. [23]).

Trait-based spatio-temporal niche segregation may be of particular importance in ecosystems mostly providing homogeneous flower resources constraining the ability to partition resources based on flower matching. Such ecosystems are common in hemi-boreal forests that, in spring, have an understory dominated by bilberry (*Vaccinium myrtillus*), a keystone floral species that is a primary food source for multiple species of bumblebees [24,25]. In such habitats, light is a feature that varies dramatically in both space and time because structural heterogeneity generates sharp gradients in light conditions that create both temporally and spatially varying microhabitat conditions [26]. We therefore hypothesized that bumblebee species foraging on bilberry achieve niche separation by foraging under different light intensities.

In this study, we tested the hypothesis that bumblebee species demonstrate light-related niche separation associated with their visual traits, as a potential mechanism underpinning species coexistence. We performed community composition analyses of bumblebees foraging on bilberry in hemi-boreal forest. With each bee observation, we included measurements of light and temperature to assess the potential association of these variables with species identity and traits [27,28]. We related our observations to key morphological traits including body size, which relates to both bees' eye size [22,29,30] and thermoregulatory ability [31,32]. In addition, we related observations to the eye parameter trait, which relates to the trade-off between light sensitivity and resolution that comes with facetted eyes [33]. A low eye parameter indicates an investment in resolution—which would aid in the detection of flowers in bright light—and a high value an investment in light sensitivity—which would improve vision at low light intensities [33]. The eye parameter trait has previously been associated with bumblebees' habitat preferences [2].

We first assessed how light and temperature related to bumblebee community composition. As our models indicated that light, rather than temperature, was the strongest driver of bumblebee community composition we then focused on addressing the following questions: (i) does bumblebee community composition vary with light intensity? (ii) Do bumblebees' visual traits explain community composition changes with light intensity? (iii) Do bumblebee species show consistent responses to light? (iv) Is the realized light niche optimum related to visual trait? We hypothesized that larger species would be more frequently observed foraging in darker and cooler microhabitats, in space in terms of shaded parts of the forest floor and in time in terms of twilight either because they have larger, more sensitive eyes or because they are more cold-adapted. We also hypothesized that bumblebees with higher eye parameter values, indicating high light sensitivity, would be more frequently observed foraging in the same darker microhabitats as above and vice versa.

## 2. Material and methods

### (a) Study sites

**Figure 1.**
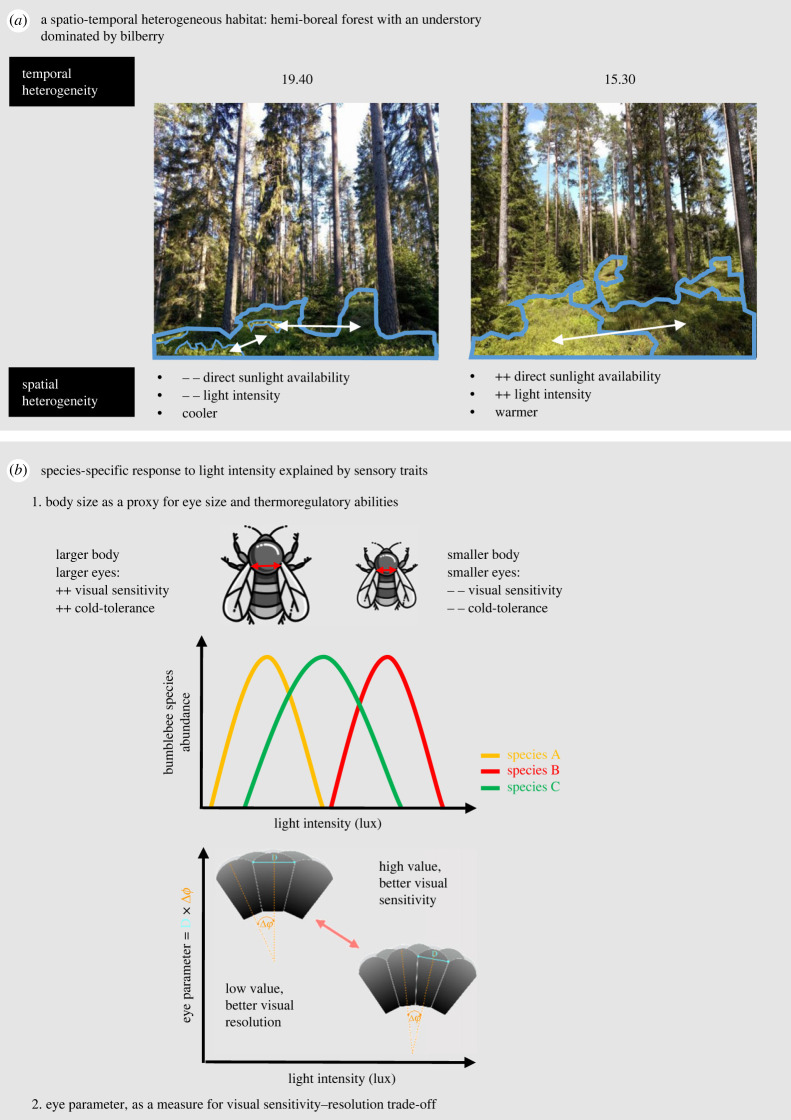
Study background, concepts and hypotheses. (*a*) Local variation light intensities in managed hemi-boreal forest with bilberry-dominated understory at one of the study sites. Forest understory dominated by shaded patches (left panel) and sunny patches (right panel), respectively. Both pictures were taken at the same site. (*b*) Predicted response to light intensity of bumblebee species with different functional traits related to hypotheses tested in the study. (1) Body size can serve as a proxy for both eye size (as eye and body size are allometrically related) and thermoregulatory ability (surface-to-volume relationship). (2) The eye parameter reflects the trade-off between resolution and sensitivity in insect compound eyes. To cope with reduced light intensity (top left), insects living in dim habitats typically have higher facet diameters (D) and higher angle between each unit (Δφ) than their bright light counterparts (bottom right). To get more light in (increased D), dim-adapted eyes necessarily reduce resolution (increased *Δφ*), leading to an overall increase in the eye parameter [33].

The study was performed at the research station Asa (Kronoberg county, Sweden) (57°10' N, 14°47' E) in two experimental forest sites (at 200 and 250 m.a.s.l., respectively) used for bilberry monitoring as part of a long-term study of phenology [34]. The sites were dominated by Norway spruce (*Picea abies*) and Scots pine (*Pinus sylvestris*), with occasional downy birch (*Betula pubescens*) and juniper (*Juniperus osteosperma*) (figure 1*a*). The understory was dominated by the European bilberry (*Vaccinium myrtillus*), which was interspersed by lingonberry (*Vaccinium vitis-idaea*) in one of the sites.

### (b) Bumblebee observations and environmental variables

**Figure 2.**
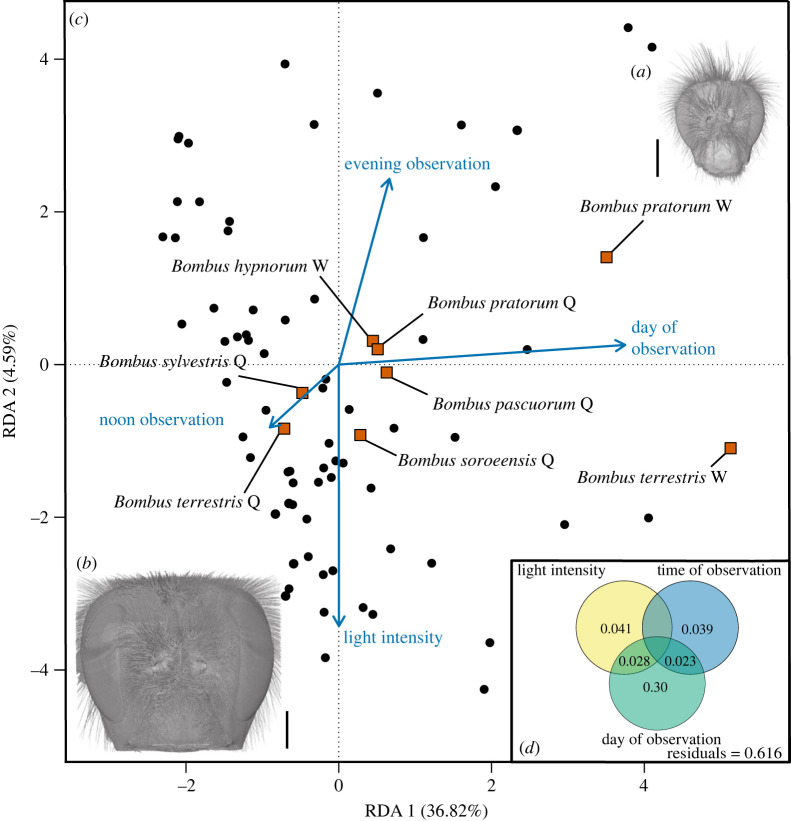
Role of light and temperature on bumblebee community composition. Volume renderings of the heads of (*a*) a *Bombus pratorum* worker, a small bumblebee species with a high eye parameter and (*b*) a *B. terrestris* queen, a large bumblebee species with a low eye parameter. Both scans are on scale. The black vertical line stands for 1 mm. (*c*) RDA of the most parsimonious model after forward selection with the bumblebee species, site identities and environmental variables. Species are represented by orange squares; W and Q stands for workers and queens, respectively. The environmental variables are evening observation (after 19.00), noon observation (between 12.00 and 15.00), day of observation (Julian day) and light intensity (log-transformed lux) and are represented by blue arrows. The black dots represent transect data. (*d*) Venn's diagram of the variance partitioning of the most parsimonious RDA model. All effects in the variance partitioning—both simple and conditional—were significant at *p* < 0.01. Values are adjusted explained variation ($R_{\mathrm{adj}}^{2}$).

In each forest site, a fixed transect corresponding to approximately 20 min at a regular walking speed (or 1 h when surveying bumblebees) was set up. Transect walks were performed during the bilberry blooming peak from 18 May to 4 June 2021. Each sampling day, the transect was walked for 1 h at three occasions: at noon, when the sun was close to the zenith (between 12.00 and 15.00), in the afternoon (between 15.00 and 18.00) and at evening (from 19.00 to just after sunset, which was at 21.19 on 18 May and at 21.47 on 4 June). When the bumblebee activity was very high, the transects could not be walked in their entirety. We aimed to record bees over a wide range of light intensities, and thus only rainy weather conditions were excluded. During each transect walk, all observed bumblebees were counted [35]. As far as possible, observed bumblebees were identified to species and caste based on Söderström *et al.* [36]. Because we could not reliably distinguish *Bombus terrestris*, *B. lucorum*, *B. magnus* and *B. cryptarum,* they were collectively recorded as *B. terrestris* complex (BTC) [37]. As, at the species level, we did not have precise behavioural observations for between 8% and 20% of individuals, we chose to not include them in the analyses. For each individual insect observation, we recorded day of observation (Julian day), light intensity (digital lux meter horizontally oriented, Mastech, resolution 0.01 lux) and temperature (Skywatch Atmos, resolution 0.1°C) (electronic supplementary material, table S1). As light intensity is inherently multiplicative (perceived on a logarithmic scale by animals, fig. 2 in O'Carroll & Warrant [38]), we considered the log-transformed values of light intensity in the analyses.

### (c) Bumblebee functional traits

We predicted that three functional traits would reflect spatio-temporal variation in bumblebee communities—body size, eye size and eye parameter. Body size, measured as inter-tegular distance (ITD, in mm), was assumed to be related to bumblebee thermoregulatory ability, with larger individuals being more cold tolerant [31,32]. Body size relates allometrically to eye size (on a log–log scale, *R*^2^ = 0.91 for eye area and 0.95 for eye volume, respectively; [30]; figure 1*b*), with larger individuals thus having a higher light sensitivity. For workers, ITD measurements were taken from Kendall *et al.* [39], except for *B. soroeensis* and *B. subterraneus,* where information was taken from Streinzer & Spaethe [40] and del Castillo & Fairbairn [41], respectively. For queens, we used ITD measured on 10 queens per species on individuals from the collections of the Biological Museum Lund University (R Carrié 2022, unpublished data), except for *B. sylvestris* where information was from Kendall *et al.* [39] (electronic supplementary material, table S2). For the BTC, we averaged ITD for *B. terrestris* and *B. lucorum.* Eye parameter was included as a specific visual trait, as it reflects the compromise between investing in light sensitivity over visual resolution (figure 1*b*). This choice was supported by a fourth-corner analysis, where the eye parameter was significantly correlated to light intensity, while the inter-ommatidial angle and the facet area were not. To obtain the eye parameter for different species [2], we used measurements taken on bumblebee specimens from the Biological Museum at Lund University collected in Scania and Abisko (Sweden), as well as from specimens previously analysed in Taylor *et al.* [30]. For each specimen, the left eye was prepared as described in Taylor *et al.* [30]. Samples were scanned using X-ray micro-CT at the Diamond-Manchester Imaging Beamline I13-2 [42,43] at the Diamond Light Source, UK (beamtime numbers: MT13848, MT16052, MT17632-1, MT20385). Volumetric and computational analyses were performed as described in Jezeera *et al.* [44] (e.g. examples of head scans in figure 2*a,b*). We estimated eye parameter for all castes except queens of *B. soroeensis*, *B. hypnorum* and *B. lapidarius* for which measurements were not available. Since differences in eye parameter between castes within a species are smaller than those between species [2], we used the values of workers for these missing measurements. For the BTC complex, we averaged the data available for the two dominating species *B. terrestris* and *B. lucorum* (electronic supplementary material, table S2). As no data were available for the cuckoo species we observed (*B. sylvestris*), we used the average eye parameter of three other cuckoo species available in the dataset (electronic supplementary material, table S2), as the eye parameters of parasitic bumblebee species are significantly different from those of non-parasitic species [2]. Trait values and associated sample sizes are provided in the electronic supplementary material table S2.

### (d) Statistical analyses

All analyses were performed using R version 4.2.0 [45]. We focused on the responses of bumblebee community composition and individual species presence/absence to light and temperature gradients and if these relationships could be explained by bumblebee traits. In the analyses, the data were aggregated to the transect walk level (i.e. 1 h of observation at a particular site). Light intensity and temperature were calculated as the average light intensity and temperature of individual bumblebee observations during the transect walk.

#### (i) Do light and temperature influence bumblebee community composition?

We used multivariate statistics to describe variation in community composition. To guide the analysis, we first applied a detrended correspondence analysis (DCA) on the community matrix of the abundance of the bumblebee species (function ‘decorana', vegan package) [46]. The length of the first DCA axis was < 4 (3.03) [47], indicating a strong linearity. We therefore applied a redundancy analysis (RDA) with the function ‘rda' (*vegan* package) to determine the main environmental parameter(s) associated with bumblebee community composition [46]. In RDA, the ordination is constrained by the environmental variables, which were: (1) light intensity (continuous variable, log-transformed, lux); (2) temperature (continuous variable,°C); (3) day of observation (as a measure of seasonality, discrete variable, Julian day); (4) time of observation (categorical variable: noon (12.00–15.00) afternoon (15.00–18.00), evening (after 19.00) and (5) study site identity (categorical variable: site 1, site 2). We first built a full model including the five environmental variables (model 1). To identify the most parsimonious model, i.e. the one with the lowest Akaike information criterion corrected for small sample size (AICc, Hurvich & Tsai [48]), we used the ‘ordistep' function in the package *vegan* [46] with a forward selection (model 2). We checked for multicollinearity between environmental variables of models 1 and 2 using the variance inflation factor (VIF) from the ‘vif.cca’ function in the package *vegan* [46]. For each model, we tested the significance of the axes and terms with an ANOVA (‘anova.cca' function in package *vegan*). We focused on the results of model 2 for the subsequent analyses. To determine the relative importance of each environmental variable from this model, and thus the variable explaining most of the variation of community composition, we partitioned variation using the function ‘varpart' in the package *vegan* and illustrated it by a Venn diagram. We tested for the significance of both simple and condition effects of the variance partitioning through model comparisons with an ANOVA and 999 Monte Carlo permutation tests. As temperature was not included in the most parsimonious model, we focused our subsequent analyses on the role of light intensity in explaining community composition.

#### (ii) Does bumblebee community composition vary with light intensity?

To assess the relationships between bumblebee community composition and light intensity, we used non-metric multidimensional scaling (NMDS) (cf. [49]). NMDS is an unconstrained ordination, which means that the species ordination is determined by the species data and not by environmental variables. We used the ‘metaMDS' function from the *vegan* package based on the Bray–Curtis dissimilarity measure which is designed for count data [46]. We separately aligned the first dimension of the two-dimensional NMDS with the light intensity with the ‘MDSrotate' function, in which relationships were assessed with a Shepard diagram (function ‘stressplot') in the package *vegan* [50]. Next, we fitted a quadratic trend surface to the light intensity with the function ‘ordisurf' in the package *vegan* overlaid on the NMDS plot. Therefore, the light intensity surface which is overlaid onto the NMDS ordination does not influence the ordination itself.

#### (iii) Do bumblebees’ visual traits explain community composition changes with light intensity?

To quantify the community response to different light intensities in terms of variation between species in their visual traits [51], we calculated the community-weighted mean (CWM) of the ITD and the eye parameter for each transect, i.e. the average trait value weighted by the abundance of each species/caste *i*: ${\mathrm{CWM}}_{h}=\sum_{i\;=1}^{S}a_{ih}\times \;t_{i}\;$ with *a_ih_* the abundance of species *i* during 1 h of observation *h*, *t_i_* the trait value of the caste/species *i*. To see if variation in community composition was explained by visual traits, we related CWM to light intensity at the individual transect walk level using a linear models with CWM (ITD and eye parameter) as response variable and average light intensity as explanatory variable [52]. We used the function ‘lm’ in the package *stats* [45].

#### (iv) Do bee species show consistent responses to light?

To characterize the responses of individual bumblebee species to light intensity, we built presence/absence Huisman–Olff–Fresco (HOF) models [53] using the *eHOF* package [54]. HOF models include a hierarchical set of response models of increasing complexity (model I: constant, no trend; model II: sigmoid trend with maximum equal to the upper bond; model III: sigmoid with maximum below the upper bound; model IV: unimodal symmetrical response; model V: unimodal skewed response; model VI: bimodal symmetric response and model VII: bimodal skewed response) [53,54]. We excluded the bimodal response models that are not expected along different light intensities and might result from outliers [55]. We bootstrapped models 1000 times and selected the most parsimonious model based on AICc [48].

#### (v) Is the realized light niche optimum related to eye trait?

To understand if the light niches of different species are partitioned according to their eye parameter, their realized niche optima (RNO) were determined from the HOF models as the point or range of light intensities where the probability of species presence was the highest [54]. Values describing RNO retrieved from the function ‘Para' from the *eHOF* package [56,57] were related to species traits (eye parameter and ITD) using linear models (function ‘lm' in the package *stats* [45]). We fitted both linear and logarithmic regression lines, but focus on the former, which had better fits (highest *R*^2^). The RNO parameter is not available for HOF models of type I (i.e. with no trend).

## 3. Results

We recorded nine bumblebee species (including the BTC) over the 10 and 12 sampling rounds at the two sites, respectively. During 67 one-hour transect walks, we made 2596 individual observations (1100 queens, 1342 workers and 154 individuals not attributed to a caste), with between 10 and 113 observations per transect walk. For further community analyses, we removed 310 observations which could not be identified to the species level and a further 13 observations for which the caste was not identified. The community was dominated by BTC (51.7% of observations, 242 queens and 934 workers), followed by *B. pratorum* (20.8%, 187 queens and 287 workers), *B. soroeensis* (10.6%, 234 queens and eight workers), *B. pascuorum* (7.8%, 169 queens and eight workers), *B. sylvestris* (3.8%, 87 queens), *B. hypnorum* (3.5%, eight queens and 72 workers), *B. hortorum* (1.1%, 23 queens and two workers), *B. subterraneus* (0.3%, five queens and two workers) and *B. lapidarius* (0.2%, five queens). For §3a, b and d, we focused the analyses on the eight most abundant species and caste combinations (i.e. with a relative abundance >1%). For §3c, we included all individuals for which species and caste were identified.

### (a) Do light and temperature influence bumblebee community composition?

In the full RDA model (model 1), the constrained variables (light intensity, temperature, day of observation, time of observation and study site) explained 44.0% of the total variation. The RDA 1 axis was significant (*p* < 0.001) and accounted for 85.9% of the explained variance. Axes 2–6 were not significant (*p* > 0.1). Light intensity (*p* = 0.02), temperature (*p* = 0.001) and day of observation (*p* = 0.001) were statistically significant. Sampling site identity (*p* = 0.08) and time of observation (*p* = 0.09) were marginally significant. Since the VIF analysis did not indicate multicollinearity (all values less than 10 [58]), all environmental variables were kept in model 1.

After backward and forward selection, the most parsimonious model (model 2) included the day of observation, light intensity and the time of observation (figure 2*c*). We present here the results of the forward selection. These three constrained variables (light intensity, time of observation and day of observation) explained 42.1% of the variation. The RDA1 and RDA2 axes were statistically significant (*p* = 0.001 and *p* = 0.04, respectively) and explained 87.4% and 10.9% of the explained variance, respectively. The day of observation (*p* = 0.001), light intensity (*p* = 0.008) and time of observation (*p* = 0.02) were statistically significant. The VIF values were all below 5.2, indicating low collinearity. The absence of temperature in the most parsimonious model suggested that light was a stronger driver of community composition than temperature.

For the parsimonious model, the variance partitioning showed that the day of observation explained 30.2% of the observed variation while light intensity explained 4.1% and time of observation 3.9% (figure 2*d*). The effect of day of observation was driven by two species and castes: workers of BTC and *B. pratorum* (figure 2*c*), which scores on the first RDA axis were, respectively, 5.13 and 3.50, while all other species had scores between −0.71 and 0.63. Considering that community composition was not significantly explained by temperature and that day of observation was important for only two species and caste combinations, we focused our subsequent analyses on the effects of light and visual traits alone (but see the electronic supplementary material, figures S1–S3 for a full analysis of the temperature effects).

### (b) Does bumblebee community composition vary with light intensity?

**Figure 3.**
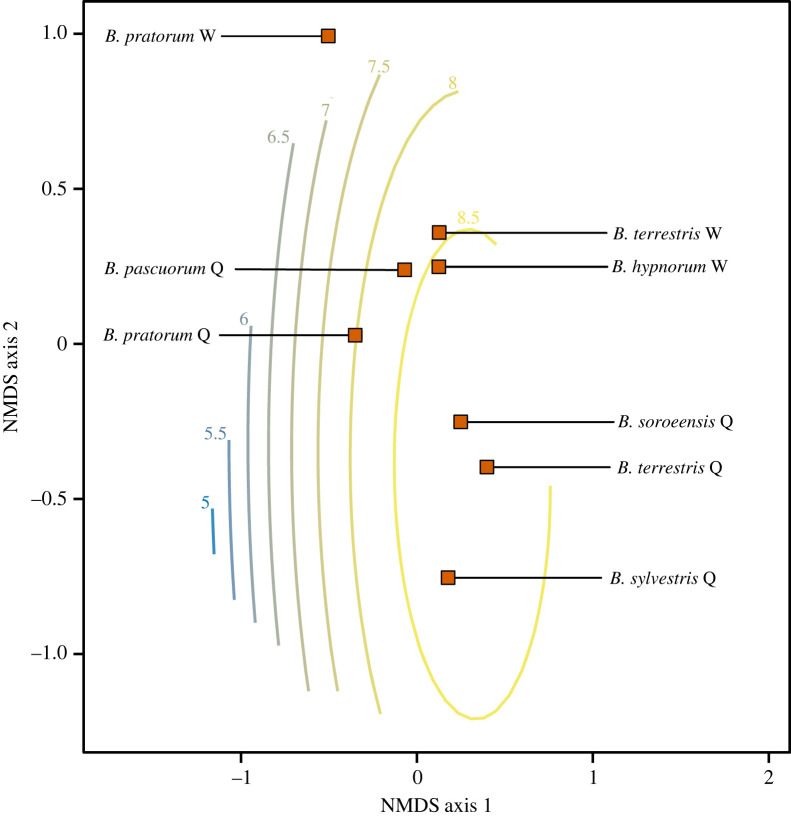
Bumblebee community segregating along light intensity levels. Bumblebee species and caste along a light gradient using non-metric dimensional scaling (NMDS) analysis. Non-metric goodness-of-fit of the ordination: *R*^2^ = 0.97. The trend surface of light intensity (log-transformed lux) (blue-to-yellow lines) was overlaid on the species NMDS (*F* = 5.12, e.d.f. = 2.41, *p* < 0.001, *n* = 67). Species are represented by orange squares. W and Q stands for worker and queen, respectively.

Bumblebee community composition was significantly associated with light intensity, with the trend surface explaining 21.8% of the observed deviance (figure 3). The stress of the two dimension NMDS was 0.17—indicating that dissimilarities are well preserved [59].

### (c) Do bumblebees' visual traits explain community composition changes with light intensity?

**Figure 4.**
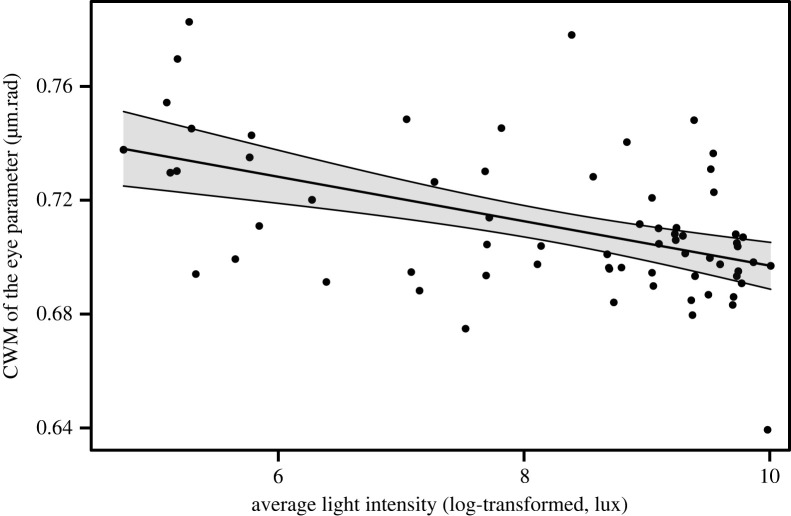
Community-level eye parameter response to light intensity levels. Relationships between the community weighted mean (CWM) of eye parameter (μm.rad) and the light intensity (log-transformed, in lux) (*F*_1,65_ = 20.70, *p* < 0.001, $R_{\mathrm{adj}}^{2}=0.23$, *n* = 67). The shaded zone represents the 95% confidence interval. The black points are the CWM of each transect walk.

The CMW of ITD was not related to light intensity (linear model: *F*_1,65_ = 1.98, *p* = 0.16, $R_{\mathrm{adj}}^{2}=0.01$, *n* = 67). By contrast, the CMW of eye parameter was significantly higher in lower light intensities (linear model: *F*_1,65_ = 20.70, *p* < 0.001, $R_{\mathrm{adj}}^{2}=0.23$, *n* = 67; figure 4).

### (d) Do bumblebee species show consistent responses to light?

**Figure 5.**
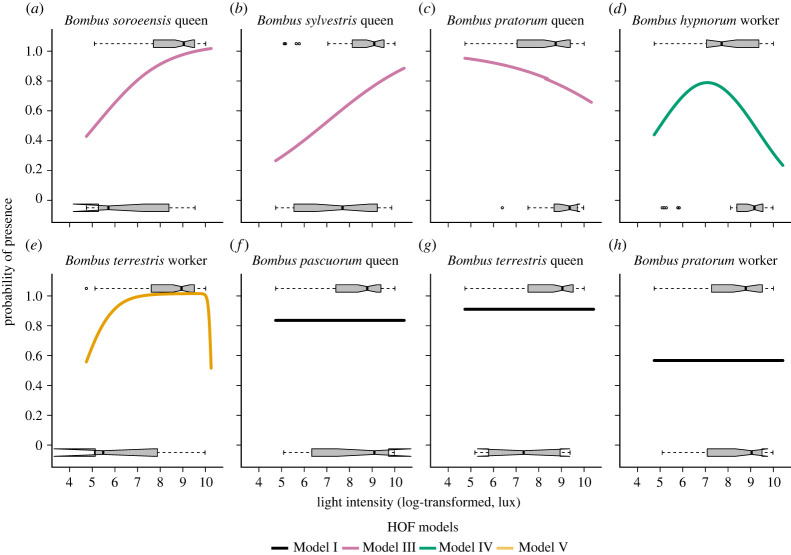
Species-specific response to changes in light intensity. Response of the most abundant bumblebee species and castes to the light intensity gradient based on occurrence data, using HOF models. The box plot indicates the distribution of the presence (*y =* 1) or the absence (*y* = 0). HOF model I (black line): constant, no trend; HOF model III (pink line): sigmoid with maximum below the upper bound; HOF model IV (green line): unimodal symmetrical response and HOF model V (yellow line): unimodal skewed response.

The HOF modelling indicated that the effect of light intensity on presence/absence varies across the eight bumblebee species/caste combinations. *Bombus soroeensis* and *B. sylvestris* queens had a probability of the presence increasing with light intensity, with a maximum at the highest recorded light intensity (HOF model II) (figure 5*a,b*). *Bombus pratorum* queens had a decreasing probability of the presence with light intensity (HOF model II) (figure 5*c*). *Bombus hypnorum* workers had a hump-shaped response, with a probability of the presence that peaked at intermediate values of light (model IV) (figure 5*d*). BTC workers had a probability of presence that increased at mid-range light intensities but that decreased at both the low and high light extremes (HOF model V) (figure 5*e*). *Bombus pascuorum* and *B. terrestris* queens as well as *B. pratorum* workers had a similar probability of being present at all light intensities (HOF model I, no trend) (figure 5*f–h*).

### (e) Is the realized light niche optimum related to visual traits?

**Figure 6.**
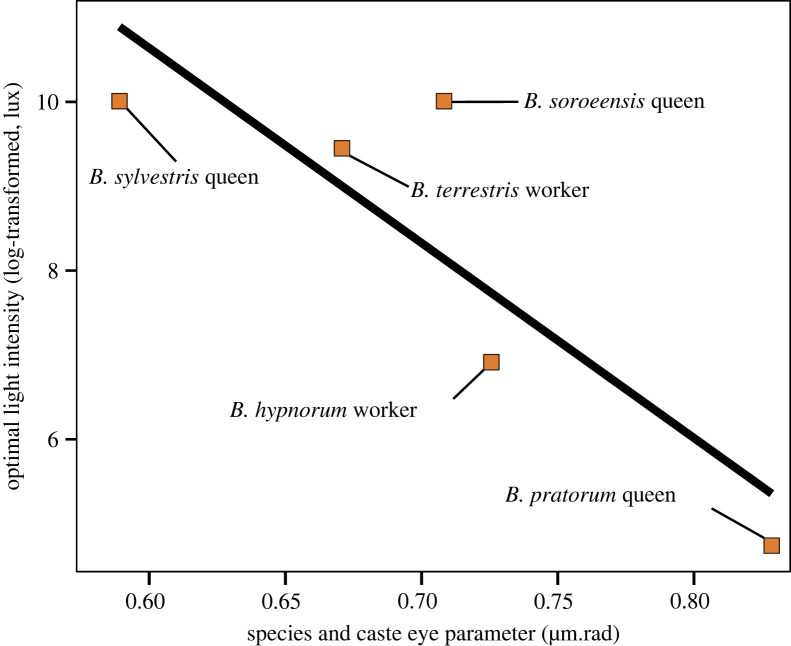
Species-specific realized light intensity niche optima in relation to eye parameter. Relationship between the optimal light intensity of bumblebee species and caste niche as derived from the HOF models and their average eye parameter (*F*_1,3_ = 1.36, *p* = 0.059, $R_{\mathrm{adj}}^{2}=0.66$, *n* = 5). Species are represented by orange squares. The black line is the regression line between realized niche optimum and eye parameter.

We found no relationship between the realized light niche optima for species/caste combinations and ITD (linear model: *F*_1,4_ = 0.29, $R_{\mathrm{adj}}^{2}=-0.21$, *p* = 0.62, *n* = 5). As the average eye parameter for species/caste combinations increased, the species' realized niche optimum tended to decrease, but this relationship was not significant (linear model: *F*_1,3_ = 8.63, $R_{\mathrm{adj}}^{2}=0.66$, *p* = 0.06, *n* = 5; figure 6).

## 4. Discussion

In this study, we combined community composition analyses and trait measurements to understand the mechanisms underlying species coexistence. Ranta & Vepsäläinen [60] proposed spatio-temporal resource heterogeneity as a mechanism that promotes coexistence of northern bumblebee species. In our study, the flower resource was indeed extremely homogeneous, providing an opportunity to identify other more subtle sources of niche partitioning. Our results suggest that bumblebee species niche partition in relation to light microhabitat conditions, which is consistent with the hypothesis of Ranta & Vepsäläinen [60], but takes place at smaller spatial and temporal scales than they suggested—which were the seasonal succession of flower resources over the flight range of bumblebee species.

Contrary to our first hypothesis, however, body size was not related to light niche. Instead, we found that bumblebee species with a low eye parameter had increasing abundance with increasing light intensity, while the abundance of bumblebee species with a high eye parameter peaked at intermediate light levels or even decreased with light. Moreover, realized light niche optima correlated with species' eye parameter. Thus, we provide evidence that bumblebee communities partitioned along light intensity in relation to species and caste combinations' eye parameters. Our findings demonstrate the importance of using sensory traits to understand how bees use their environment. More generally, our results add to a growing body of literature that highlights the importance of the association between insect sensory traits and microhabitat use, with implications for the possible role of microhabitat niche partitioning related to sensory trait expression as a mechanism underpinning species coexistence.

One way to increase visual sensitivity is to increase the size of body, as this would allow for the development of larger eyes that can catch more light [15,61]. Indeed, previous studies have observed that larger diurnal bees are more likely to forage in shaded areas than smaller bees [8,62]. As sun radiation affects both light intensity and temperature, however, a relationship between body size and light environment will not conclusively indicate reasons for niche partitioning because body size serves as a proxy for both thermoregulatory abilities [31,32] and eye size [22,30]. Interestingly, in this study, the light dimension was not correlated to body size, and it was only weakly related to temperature (*R*^2^ = 0.10, electronic supplementary material, figure S2). However, our finding that the eye parameter was significantly associated with the light dimension of the niche, suggests that light can be a driving force behind bumblebee niche partitioning. Nonetheless, it should be noted that our results do not entirely rule out the importance of temperature, since traits other than body size, such as hairiness, may affect thermoregulation [63].

For insects to be able to efficiently move and forage in areas with low light, high visual sensitivity—as evidenced by larger eyes or a higher eye parameter—is required. We found that the association of some bumblebee species (*B. pratorum*, *B. hypnorum* and to a lesser extent *B. pascuorum*) with low light levels was related to their species-specific eye parameter, which was high compared with the other *Bombus* species in the study. This suggests that these species in evolutionary terms invested preferentially into light sensitivity at the cost of visual resolution. Small-bodied species such as *B. pratorum* and *B. pascuorum*, which are predicted to have small eyes due to allometric scaling, nonetheless had high eye parameters. This investment in sensitivity might represent an adaptation for counteracting the sensitivity limitations imposed by body and eye size in these species. Indeed, similar adaptations have previously been described in the small stingless bee *Trigonisca pipioli* which had a lower light threshold than expected by its body size [61].

An intriguing question that remains is why do species with higher eye parameters forage less frequently under brighter conditions? While diurnal variation in foraging time between bumblebee species might reflect diurnal variation in nectar availability [11,64], this is unlikely to be the case with a homogeneous resource such as bilberry. Furthermore, bumblebee species were observed during the whole period of observations suggesting that nectar availability was constant and new bilberry flowers open throughout the day, providing newly available nectar from sunrise to sunset (O.B., personal observation). We therefore suggest that variation in light niche is a mechanism that reduces inter-specific competition, because bees with higher eye parameters have a *relative* foraging advantage in dim light conditions. To properly investigate the presence and/or effect of competition in bumblebees, it would be necessary to compare the realized niche of species in the presence and absence of other species, e.g. through species removal (cf. [65,66]). Further investigations of individual light sensitivity could also highlight potential intra-specific variation in habitat preferences.

In our analyses, we did not account for the effect of day of observation, as the timescale was too short to account for seasonal effects that would influence the relative proportion of queens and workers. To disentangle variation within days from variation across seasons, future studies should investigate bumblebee communities foraging in hemi-boreal forest later in the season, e.g. during the blooming of lingonberry (*Vaccinium vitis-idaea*). Moreover, variation in community composition related to visual traits could be driven by other correlated traits such as olfactory or physiological traits, which were not measured in this study and which might explain why some species did not segregate along the light gradient. One limitation of our data is the low number of eye parameter measurements per species and caste due to the time-consuming nature of acquiring these data [30,44]. Nonetheless, in bumblebees eye parameter is more variable between species than between castes within a species [2]. Further experiments thoroughly disentangling light intensity and temperature while controlling for thermal resilience are required to explore the mechanisms explaining species coexistence. Finally, it would be worth further investigating the potential microhabitat niches and visual differences of the species of the *BTC*, as *B. lucorum*, *B. magnus* and *B. cryptarum* were shown to have distinct climate and habitat preferences [67,68]. However, the inclusion or exclusion of this species complex from the community analyses did not change the role of light intensity for community composition.

Overall, this study provides an explanation of how different species of bumblebees coexist when foraging on the same homogeneous resource—they exploit spatial and temporal fluctuations in light intensity to exploit particular light niches. We show that this niche partitioning is related to a visual functional trait rather than body size. In more general terms, our work demonstrates the importance of exploring visual traits as a mechanism explaining habitat choice and niche partitioning. This highlights the potential of sensory traits to understand how changes in habitat, for example, those caused by field abandonment, afforestation and climate-related tree-line shifts which result in changes of light environments, may affect the community composition of diurnal pollinators.

## Data Availability

The datasets and scripts supporting this article are available in the electronic supplementary material. The data are provided in electronic supplementary material [69].
